# Carbon Dots: An Innovative Tool for Drug Delivery in Brain Tumors

**DOI:** 10.3390/ijms222111783

**Published:** 2021-10-29

**Authors:** Giovanna Calabrese, Giovanna De Luca, Giuseppe Nocito, Maria Giovanna Rizzo, Sofia Paola Lombardo, Giulia Chisari, Stefano Forte, Emanuele Luigi Sciuto, Sabrina Conoci

**Affiliations:** 1 Dipartimento di Scienze Chimiche, Biologiche, Farmaceutiche ed Ambientali—Università degli Studi di Messina, Viale Ferdinando Stagno d’Alcontres, 31, 98168 Messina, Italy; giuseppe.nocito@unime.it (G.N.); mgrizzo@unime.it (M.G.R.); sabrina.conoci@unime.it (S.C.); 2 Istituto Oncologico del Mediterraneo, Via Penninazzo 7, 95029 Viagrande, Italy; sofia.lombardo@grupposamed.com (S.P.L.); giulia.chisari@grupposamed.com (G.C.); 3 IOM Ricerca, Via Penninazzo 11, 95029 Viagrande, Italy; stefano.forte@grupposamed.com; 4 A.O.-Universitaria Policlinico “G. Rodolico–San Marco”, Via Santa Sofia 78, 95123 Catania, Italy; e.l.sciuto@gmail.com

**Keywords:** brain tumors, drug-delivery systems, blood-brain barrier, nanoparticles, carbon dots, nanocarriers, theranostic

## Abstract

**Simple Summary:**

The pharmacological treatment of tumors of the central nervous system poses major challenges due to the presence of physical obstacles, i.e., the blood-brain barrier, impeding the delivery of anticancer drugs to the tumor site. Hence, the development of innovative therapeutic strategies to overcome these obstacles is of pivotal importance to reach significant clinical advances in brain tumor treatment. In this review, we report the latest studies on carbon dots as an innovative tool for brain tumor drug delivery.

**Abstract:**

Brain tumors are particularly aggressive and represent a significant cause of morbidity and mortality in adults and children, affecting the global population and being responsible for 2.6% of all cancer deaths (as well as 30% of those in children and 20% in young adults). The blood-brain barrier (BBB) excludes almost 100% of the drugs targeting brain neoplasms, representing one of the most significant challenges to current brain cancer therapy. In the last decades, carbon dots have increasingly played the role of drug delivery systems with theranostic applications against cancer, thanks to their bright photoluminescence, solubility in bodily fluids, chemical stability, and biocompatibility. After a summary outlining brain tumors and the current drug delivery strategies devised in their therapeutic management, this review explores the most recent literature about the advances and open challenges in the employment of carbon dots as both diagnostic and therapeutic agents in the treatment of brain cancers, together with the strategies devised to allow them to cross the BBB effectively.

## 1. Introduction

Cancer represents one of the leading causes of death worldwide. Brain tumors, frequently defined as brain neoplasms, are a heterogeneous group of neoformations affecting the central nervous system (CNS). Even if less common than other cancer types, CNS tumors are particularly aggressive and represent a significant cause of morbidity and mortality in adults and children [1]. They affect the global population and are responsible for 2.6% of all cancer deaths, 30% of children’s, and 20% of young adults’ [2,3]. Brain tumors can be firstly classified based on their benign or malignant character, then on their origin as primary if they arise from CNS cells, and as secondary or metastatic if they start as metastases of other cancers [4]. Gliomas are the most common malignant brain tumors and comprise astrocytomas, oligodendrogliomas, ependymomas, and several uncommon histologies [5]. According to the World Health Organization (WHO) criteria, they are histologically classified into four grades (I–IV) related to cancer morphology, biology, and prognosis.

Glioblastoma (GB) accounts for 50–60% of all gliomas and is a grade IV astrocytoma. Its prognosis is the worst, with a 12–15 months’ average survival after diagnosis [6,7]. Glioblastoma is characterized by specific morphological features, such as increased cellularity, conspicuous nuclear atypia, the abundant mitotic activity of cancer cells, neo-angiogenesis, and tumor necrosis. To date, the standard glioblastoma treatment comprises invasive reduction of the tumoral mass, radiotherapy, and chemotherapy with temozolomide [8]. Failure in its treatment is mainly due to the incapacity to surgically eradicate the whole tumor mass, the absence of effective drugs able to cross the BBB, and the growth of multidrug resistance, which contributes to cancer metastasis and patient relapse.

Malignant cancer management requires understanding those characteristics of both the tumor and its microenvironment that significantly influence therapeutic response and clinical outcome. In this context, nanomaterial-based anticancer therapies have become attractive as a means of controlling the tumor microenvironment (TME) and they have the potential to exceed conventional treatments [9,10,11]. The growing interest in the application of nanotechnology in cancer medicine, referred to as cancer nanomedicine, is mainly due to its promising applications, including drug delivery, in vitro diagnostics, in vivo imaging, and targeted therapy. Currently, a wide variety of therapeutic nanoparticle (NP) platforms, including lipid-based, polymer-based, inorganic, viral, and drug-conjugated nanoparticles (NPs), have been approved for use in the clinic as nanocarriers for cancer treatment. Nanocarriers, due to specific properties such as smaller size, high surface-to-volume ratios, higher reactivity, drug release profiles, targeting modifications, as well as specific optical properties, can better reach cancer tissue and release drugs in a controlled manner [12,13]. Among the most-used nanocarriers, carbon nanomaterials, including graphene, fullerenes, carbon nanotubes, and carbon quantum dots, have attracted the attention of cancer theranostics for their optical, electronic, thermal, and mechanical properties and their versatile and biocompatible functionalization [14].

Carbon dots (C-dots) have been used as drug delivery systems (DDS) to improve drug solubility, half-life, and accumulation at the tumorous site, reduce the drugs’ side effects, and increase their bio-availability and tolerance [15,16]. In this review, we present a literature survey about the application of C-dots in brain cancer nanomedicine and highlight the progress in this field.

## 2. Brain Tumors and Therapeutic Management

There are hundreds of histologically different types of primary brain and CNS cancers. Gliomas, neuroepithelial tumors originating from the glial or supporting cells of the CNS, account for 24% of all primary brain and CNS tumors. Gliomas vary greatly in histology, from benign ependymomal tumors to the most aggressive and deadly grade IV GB. Glioblastoma is the most common malignant brain tumor, representing about 57% of all gliomas. It is slightly more diffused in males than females, and the median age at diagnosis is 65 years [17]. Recent evidence reported that GB incidence is highest globally in North America, Australia, and Northern and Western Europe [18]. GB still has a poor prognosis and some factors, including advanced age, poor performance status, and incomplete extension of the resection, contribute negatively [19]. The median survival for elderly patients who receive treatment is 15 months [20], while the survival rate is only 5.8% at five years post-diagnosis [2].

The first approach to the treatment of brain tumors is surgery. Depending on the tumor type and location, the resection could be total or partial. This latter method is preferred, especially in diffuse gliomas, to preserve brain functions and the highest quality of life possible, and an additional positive aspect lies in the removed tissue being useful for histological and molecular analyses. Unfortunately, however, the conventional approach is often non-effective. Benign, non-diffuse, and, in some cases, low-grade diffuse gliomas are successfully cured with surgical resection. In contrast, complete eradication is not possible for high-grade gliomas, such as glioblastoma multiforme. The outcome is frequently poor in these cases, only aiming for a short increase of patient life expectancy with the help of chemotherapy and radiotherapy [21]. Moreover, despite the great efforts being made to increase patient survival, current treatments for high-grade gliomas lack efficacy, partly due to the impossibility of chemotherapeutics to cross the various barriers that prevent drugs from reaching the tumor sites [22].

There are three main obstacles to brain tumor treatment: the blood-brain barrier (BBB), the blood-brain tumor barrier (BBTB), and a relatively weak enhanced permeability and retention (EPR) effect.

The BBB comprises a highly specialized endothelial cell monolayer, in part covered by pericytes and basement membrane and almost entirely surrounded by the end-feet of astrocytes. It forms a heavily restricting barrier that maintains the CNS homeostasis by limiting the passive uptake of large and hydrophilic molecules and excluding toxins. Further, it strictly limits drug transport into the brain by serving as a physical (tight junctions), metabolic (enzymes), and immunological barrier [23].

In analogy to the BBB, the BBTB is located between brain tumor tissues and the capillaries formed by highly specialized endothelial cells, preventing the paracellular delivery of most hydrophilic molecules to the tumor site [24]. This barrier is formed only upon developing brain tumors when cancer cells begin to invade the surrounding normal brain tissue, and the BBB is damaged (tumor volume > 0.2 mm^3^) [25]. The BBTB in low-grade gliomas has the same barrier function as the BBB under normal conditions. On the other hand, although the BBTB is altered in high-grade gliomas, this alteration is not sufficient to allow the delivery of therapeutic quantities of drugs and, thus, represents an obstacle for brain-targeted drug delivery [26].

The EPR effect appears as the brain tumor grows. Indeed, most solid tumors possess an abnormal vascular production sustained by high levels of vascular growth factors, like vascular-endothelial growth factor and vascular permeability-enhancing factors (e.g., bradykinin, prostaglandin, nitric oxide), together with a lack of functional lymphatic drainage. These characteristics are the basis for invasive and rapid tumor growth and lead to new blood vessels with larger lumen, wider fenestrations, and an increase in fluid pressure, determining the EPR effect [27]. This effect enables the extravasation and retention of macromolecules in tumors, allowing nanocarriers with an appropriate particle size to enter the brain neoplasms via the endothelial gaps on the microvessels.

## 3. Drug Delivery Strategies for Brain Tumors

The effective treatment of brain diseases is one of the most complex challenges in oncology due to the many hurdles related to the transport of drugs to the brain. Conventional anticancer treatments are moderately successful in reducing tumor volume and metastases, due to the low permeability and high selectivity of the BBB and BBTB.

Recently, significant efforts have been made to overcome current brain cancer treatment limitations and develop novel drug delivery strategies, e.g., using nanocarriers to deliver drugs across the BBB. These include natural carriers, vesicles/liposomes, nanoparticles, and exosomes loaded with anticancer payloads [28,29].

### 3.1. Physical Drug Delivery Strategies

Treatments based on the delivery of drugs through the cardiovascular system often need high systemic drug loads to reach appropriate drug levels at the treatment site. Physical approaches have, thus, been devised to reduce the systemic toxicity ensuing from drug delivery through systemic circulation. Often, these methods involve the direct delivery of the drug into the brain interstitium or tumor parenchyma, also making use of catheter/pump systems or drug-loaded biodegradable implants, and result in the diffusion of the therapeutic agents from sites with a high drug concentration to the tumor periphery [22,30,31]. However, these are fairly invasive treatments that cause a breach in the integrity of the BBB and involve the risks of infection or brain trauma. One method that focuses on the temporary disruption of the BBB to enable the delivery of circulating agents is based on the intra-arterial injection of hyperosmotic solutions (e.g., 25% mannitol in water), but ca. 6% of the treatments still result in focal seizures [32]. A less-invasive approach to achieving temporary BBB disruption makes use of focused ultrasounds. Their effect is enhanced by injecting preformed microbubbles, commercially available as ultrasound imaging contrast agents: the gas particles concentrate the sonication effect on the brain microvasculature, causing a temporary widening of tight junctions and activating transcellular mechanisms, thus allowing CNS drug delivery [33].

### 3.2. Chemical Drug Delivery Strategies

**Figure 1:**
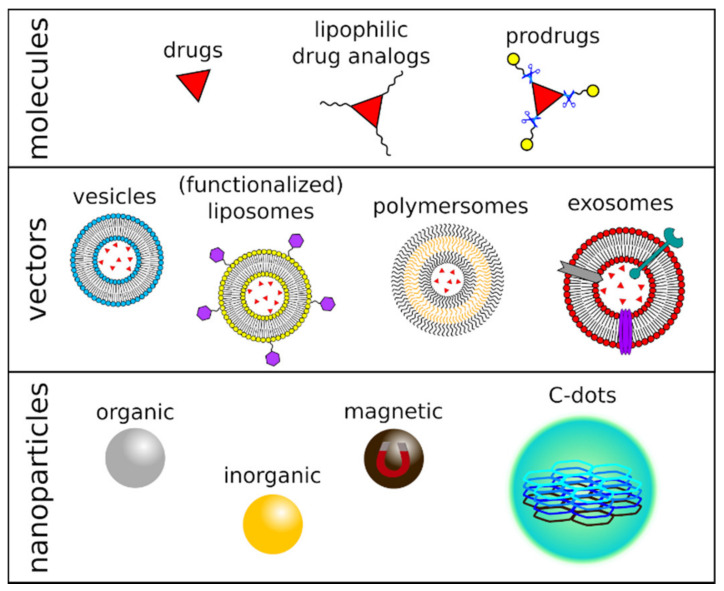
Figurative representation of chemical-, vector-, or nanomaterial-based drug delivery systems.

Concerning the chemical approaches for brain tumor-targeted drug delivery, it should be noted that only small, mildly lipophilic molecules can cross the BBB spontaneously [34], whereas hydrophilic molecules need strategies to be devised that overcome their poor cerebrovascular permeability. Straightforward approaches still exploit passive brain uptake, after a drug is converted into either its lipophilic analog or a prodrug through covalent chemistry (Figure 1). Employing a prodrug requires the molecule to be metabolized after its administration to bring about the desired anticancer activity [22]. Most recent strategies regarding prodrugs are based on dynamic covalent chemistry or supramolecular chemistry, where the prodrug is formed through reversible covalent bonds or non-covalent host-guest interactions, respectively [35,36]. These approaches are novel and exciting alternatives to their covalent counterparts, for example via more accessible synthetic protocols, while keeping the capability to protect the active species and sensitive response to biological environments, providing a high-fidelity release of drugs [36]. Nevertheless, while approaches leading to increased lipophilicity may boost drug permeation across the BBB, they also lead to enhanced drug association with plasma proteins, increased uptake into other tissues, and lower solubility into body fluids, resulting in a reduction of the available concentration of the drug and of its activity against the brain tumor [37].

### 3.3. Vector-Based Drug Delivery Strategies

Carrier-mediated drug delivery and receptor-mediated endocytosis are two strategies that exploit the endogenous BBB transport system to overcome the CNS’s natural inaccessibility to drugs. They use the transport systems within the brain capillary endothelium, most of which are passive, that take care of the brain’s uptake of nutrients, metal ions, peptides, and hormones [38]. Receptor-mediated endocytosis is activated by specific ligands that end up being transported into the CNS. Thus, these transporter ligands are covalently linked to the drug of interest to trick the molecular machinery and enable drug uptake. The type of covalent bond is conveniently selected to allow the later detachment of the drug to restore its pharmacological activity [39]. For example, transferrin receptor- and insulin receptor-mediated endocytosis systems have been used for small molecules and therapeutic protein delivery [40,41,42]. Several polar drugs can rapidly and selectively cross the BBB via carrier-mediated transport, including carriers for glucose (GLUT1), monocarboxylic acids (MCT1), large neutral amino acids (LAT1), cationic amino acids (CAT1), and nucleosides (ENT 1-2, CNT1-2) and choline [43].

Another family of CNS transporters includes the ATP-binding cassette (ABC) forms. These consist of 48 transporting proteins, classified in subfamilies from A to G, that regulate the influx of nutrients and small molecules and the activity of the intracellular organelles. They are also involved in multidrug resistance, given their activity as drug effluxers, i.e., they reject the permeated drugs from intracellular compartments or, in the case of the CNS, from the internal compartment to the systemic blood circulation. Targeting ABC transporters involved in drug efflux could, in principle, constitute a valuable strategy to enhance drug activity in the CNS [44,45].

Vesicles, particularly liposomes and polymersomes, have also been employed as a DDS targeting brain tumors (Figure 1). These nano- or micro-sized self-assembled structures are synthetic models for cell membranes and consist of natural or synthetic surfactants, lipids, or amphiphilic block copolymers [46]. These molecular components share the same structure: they all present a polar head and a hydrophobic tail, assembled into single- or double-layered supramolecular membranes. The vesicles are, thus, capable of incorporating hydrophilic, lipophilic, and hydrophobic drugs in their different compartments and present many advantages as a DDS, e.g., biocompatibility, low toxicity, and controlled release of the therapeutic agents. Additionally, their surface can be modified via the inclusion of peptides, polysaccharides, or antibodies, to name a few macromolecules, to improve brain-specific delivery and act as efficient diagnostic or therapeutic tools against brain tumors [47]. Still, their effectiveness is limited: among the most known and used vesicles for drug delivery, pegylated liposomes accumulated into the CNS in a low amount [48] and exhibited limited clinical efficacy against high-grade gliomas [49,50,51].

Exosomes are small extracellular vesicles secreted by cells exhibiting exciting features, including stability, biocompatibility, permeability, low toxicity, low immunogenicity, and an almost endless variation in loading and homing abilities, representing a promising DDS for brain tumors. Traditional chemotherapeutics, natural drugs, as well as nucleic acid have been encapsulated in exosomes aiming at the treatment of glioblastoma. However, to date, examples of clinical trials addressing the use of exosomes as a drug carrier for solid tumors are still limited, primarily because of problems in large-scale production, quality control, and storage of these vectors [52].

### 3.4. Nanomaterial-Based Drug Delivery Strategies

In the last years, the advent of nanomedicine has diffusely presented NPs as fascinating tools able, among other things, to improve drug transport across the BBB [53]. Thanks to their structural and physicochemical characteristics, these nanomaterials present several advantages as a DDS, including ease of transporting drug payloads, ability to control drug release (fast or sustained) and pharmacokinetics, as well as good tolerability. Their high chemical and biological stability, the ability to incorporate hydrophilic and hydrophobic pharmaceuticals, and the possibility of being administered by different routes (oral, parenteral, inhalation) have made NPs ever more attractive for medical applications. Furthermore, simple methods are available to engineer the surface of these nanostructures through covalent/non-covalent (multi)functionalization, providing tissue-specific targeting capabilities after conjugation with the appropriate ligands, including antibodies, proteins, or aptamers [54].

As already mentioned, NPs can penetrate the tumor tissue and deliver their cargoes to neoplasms, thanks to the EPR effect [55], taking full advantage of the differences between normal and tumor environments to deliver therapeutics to cells [56]. As normal tissues are less inclined to grant NPs access due to their healthy vasculature, the microarchitecture of the BBB is characterized by the loss of endothelial cell tight-junction in high-grade gliomas while it is preserved in low-grade ones [57,58,59]. Therefore, NPs can accumulate in the tumor microenvironment and perform their designed cytotoxic and delivery tasks. The employment of these nanomaterials as drug delivery systems has also led to an increase of drug concentration at the surface of the BBB, offering further chances to cross it by increasing drug circulation time in the blood, compared to more conventional approaches. Still, despite the promising application of nanoparticles in anticancer drug delivery, given their theoretical ability to accumulate within tumoral sites via the EPR effect, many experiments have resulted in a poor outcome when this concept has been translated to brain tumors [44].

Notwithstanding these initial drawbacks, research has never stopped designing better pharmaceutical nanotechnologies to target the CNS and allow crossing the BBB through modification in NP size, shape, or surface functionalization [44,60]. Carbon dots are indeed among the newest, most encouraging systems that meet the targeted drug delivery requirements. They also open the way to simultaneous bio-imaging applications, thanks to their tunable luminescence that allows real-time identification of their tissue distribution.

## 4. Carbon Dots for Drug Delivery in Brain Tumors

### 4.1. Carbon Dots

**Figure 2:**
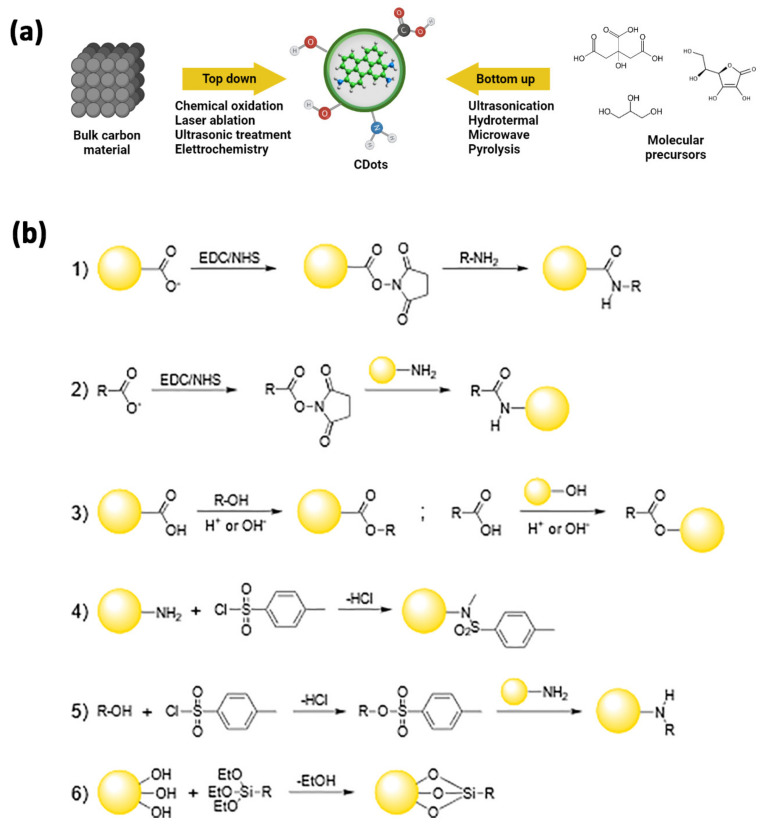
(**a**) General approaches for the synthesis of C-dots. In a top-down approach, C-dots are synthesized by transforming bulk carbon material into ultra-small powders via oxidation, laser ablation, ultrasounds, or electrochemical methods. In a bottom-up approach, C-dots are synthesized via physical or chemical treatments of molecular precursors that may get ionized, dissociated, evaporated, or sublimated and then condense/react to form C-dots, or via hydrothermal, sonochemical, pyrolitic treatments. (**b**) Reactions schemes for covalent strategies to C-dots functionalization: amide coupling (**1**,**2**), esterification (**3**), sulfonamide formation (**4**); tosylate-leaving group in nucleophilic substitution (**5**), sylilation (**6**).

Since their accidental discovery during the electrophoretic purification of arc-discharged single-walled carbon nanotubes [61], carbon dots (C-dots) [62] have gained significant interest in biomedical applications thanks to their physicochemical properties. C-dots exhibit size tuneability in the nanometer range, size-dependent photoluminescence, excellent photostability, and exceptional biocompatibility [63]. These carbon nanoallotropes consist of nearly isotropic nanoparticles having sizes below 10 nm, a nanocrystalline graphitic core with great sp^2^ character, and high oxygen content at their surface [64]. They differ notably from nanodiamond particles, which are produced under hard-to-obtain conditions and constitute a diamond (sp^3^) core with a sp^2^ carbon shell. The difference between C-dots and graphene quantum dots is, instead, mainly linked to their morphology, the first being spherical particles, while the second are better described as zero-dimensional graphene disks [65]. The synthetic strategies leading to the production of C-dots can be broadly divided into top-down and bottom-up approaches (Table 1) [66,67]: the former includes the fragmentation of starting carbonaceous materials using physical or chemical methods (e.g., electrochemical synthesis [68,69,70,71,72,73], chemical oxidation [74,75,76,77,78,79], arc discharge [61,80,81,82], and laser ablation [62,83,84,85,86]); the latter starts from molecular precursors and consists, among others, of ultrasound [87,88,89] and hydrothermal [90,91,92,93,94,95] treatments, microwave-assisted synthesis [96,97,98,99,100,101], and pyrolysis or carbonization of the reactants [102,103,104,105,106]. The surface of C-dots typically presents functional groups like carboxyl, hydroxyl, or amino groups, and their composition heavily depends on the nature of the precursors or the reaction conditions, often leading to significant differences in the nanoparticles’ properties [107,108] (Figure 2). The intrinsic presence of polar residues on the C-dots’ surface endows them with good solubility in water, which is very convenient for developing applications in nanobiology or nanomedicine [109]. Although this eliminates the need for additives to provide the NPs with an affinity for the aqueous environment, it is still desirable to modify the functional moieties at the surface of the original carbon nanomaterials, e.g., to increase their biocompatibility or impart the C-dots with new chemical functions. The natural occurrence of accessible and reactive functional groups is largely exploited both in covalent and non-covalent surface treatments [66,107,109], providing C-dots with new (bio)sensing capabilities, improved photoluminescence, broader in vitro and in vivo bioimaging, as well as drug-delivery and theranostic capabilities [13,110,111,112,113].

As briefly stated above, C-dots can be easily engineered by exploiting the covalent chemistry of all the functional groups present at their surface.

Since carboxyls are among the most abundant moieties available, the amide coupling reaction is an obvious and advantageous choice to change the surface characteristics of these NPs. This approach could be employed both to enhance their photophysical properties, which could be negatively affected by the electronic effects exerted by too many oxygen-rich groups [107], and to attach the appropriate bio-label to the C-dots. One of the most common methods makes use of the standard EDC/NHS-catalyzed reaction, yielding the coupling of surface carboxyl moieties with amine groups into an amide bond (Figure 2(b1)) [116]. Notably, the same approach may be used when starting with N-doped C-dots, whose surfaces are usually decorated by amino groups and react with carboxyl-containing targets (Figure 2(b2)) [117].

A different strategy employs esterification reactions by coupling complementary hydroxyl and carboxyl groups, one present at the C-dots’ surface and the other onto the target to be bound to the NPs (Figure 2(b3)) [118,119].

In addition, amino-functionalized C-dots can be coupled to functional moieties of interest through sulfonylation reaction, attaching the proper sulfonyl chloride compounds on their surface, via the direct formation of sulfonamide bonds (Figure 2(b4)) or by forming intermediates containing better leaving groups in S_N_2 reactions (Figure 2(b5)) [120,121,122].

Silylation reactions are also of great interest since they can lead to C-dot/silica nanohybrids. In these reactions, the alkoxy groups of the appropriate organo-functional tri-alkoxysilane may react with the surface hydroxyls of the C-dots and form Si-O-C bonds, resulting in the NPs being decorated with the organic function of interest (Figure 2(b6)) [123,124].

Non-covalent strategies for engineering the C-dots’ surface properties are based on the formation of multiple supramolecular interactions, such as electrostatic, coordination, or π bonds [107]. Electrostatic interactions may involve positively or negatively charged C-dots and small molecules, polymers, or other species having an opposite charge [125,126,127]. Exploiting complexation for the surface modification of the carbon nanoparticles starts from having functional groups capable of coordination interactions on the surface of the NPs, such as amino or carboxyl moieties. These can then bind the metal ion of interest, leading to new luminescent features in case of binding to rare-earth atoms or to sensoristic applications, to name a few [122,128,129]. Finally, the exposed aromatic portions of the C-dots surface can be involved in π interactions with small aromatic molecules, allowing them to be tuned for their photophysical properties or to act as a delivery system for aromatic drugs [130,131].

### 4.2. Carbon Dots Crossing the BBB

The BBB remains an important obstacle between the brain and compounds circulating in the blood. It functions as a blockade, avoiding the arrival of toxins and cells into the brain. Therefore, its integrity is one of the main factors necessary to maintain homeostasis and to protect this organ [132]. Notwithstanding these important functions, the BBB notably reduces drug transport into the brain via blood circulation [133]. Hence, developing a system able to cross it when necessary to deliver therapeutic and diagnostic drugs to the brain is a major challenge. In this context, recently, C-dots attracted great attention as a promising tool for drug delivery thanks to their relevant properties, including ultra-small size, low toxicity, high drug-loading capacity, long-term stability, and controlled drug release capabilities [134]. C-dots-based therapeutic approaches have shown greater facility to cross the BBB and transport and deliver neurological drugs into the CNS. In an in vivo study, Li et al. demonstrated that C-dots functionalized with transferrin could enter the CNS by crossing the BBB, indicating that they can be used to treat neurological diseases [135]. In another study, Seven et al. reported that C-dots prepared from glucose and conjugated to fluorescein cross the BBB in zebrafish and rats without needing an additional targeting ligand, pointing out their potential as a drug delivery system for the CNS [136]. Zhou et al. showed that amphiphilic, yellow-emissive C-dots could cross the BBB of 5-day-old wild-type zebrafish. These NPs could enter the cells to inhibit the overexpression of human amyloid precursor protein (APP) and β-amyloid (Aβ), which are among the main factors responsible for Alzheimer’s disease. Their results hint at these C-dots’ great potential as nontoxic nanocarriers for drug delivery toward the CNS, as well as promising inhibiting agents for Aβ-related pathologies.

### 4.3. Carbon Dots as Drug Delivery Systems in Brain Tumors Treatment

**Figure 3:**
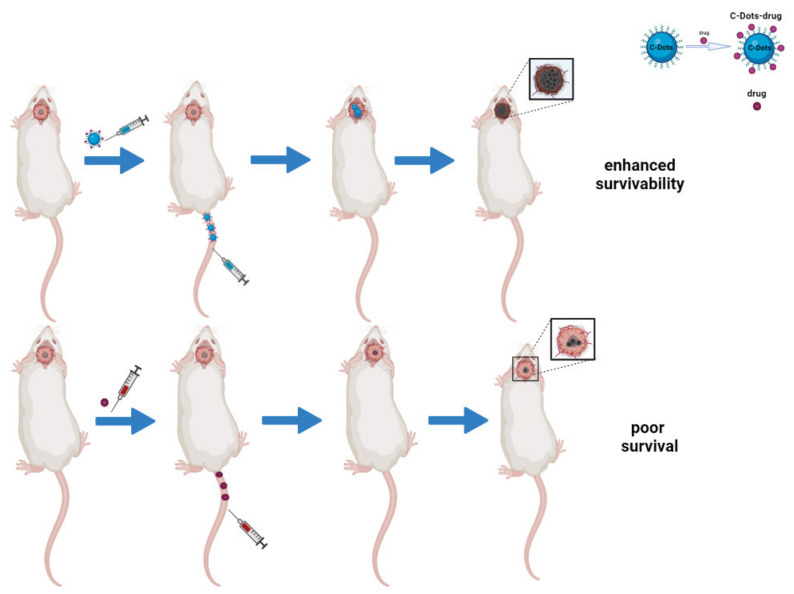
Schematic representation of C-dots as drug delivery systems in brain tumors in vivo. The image compares the efficacy of C-dots/drugs conjugates to free-drug approaches. The first system has a better therapeutic effect because of its ability to cross the BBB and inhibit tumor growth, thus enhancing survivability.

In the last few years, C-dots have been broadly synthesized, characterized, and used as promising nanocarriers for drug delivery [137], taking full advantage of favorable features such as their small size (<5 nm) and a rich surface chemistry that allows the binding of receptors and chemotherapy drugs (Figure 3) [138,139]. C-dots are reported to have increased the solubility in water, bioavailability, half-life, and tumor accumulation of drugs as part of a nano-formulation by exploiting the EPR effect [140,141]. Besides this, their localization can be easily detected thanks to their brilliant fluorescence, paving the way to theranostic applications.

Several in vitro and in vivo studies regarding the ability of C-dots and their conjugates to overcome the BBB have been reported in the literature (Table 2), garnering much interest from the scientific community [142,143,144].

Lu et al. [145] synthesized nitrogen-doped C-dots (C-dot/PEI) using a one-pot hydrothermal treatment with citric acid in the presence of Polyethyleneimine (PEI), obtaining particles with an average size of ~2.6 nm and strong blue luminescence. Upon evaluating their ability to cross the BBB by means of an in vitro model of rat microvascular endothelial cells and astrocytes, C-dot/PEI exhibited excellent photostability and low cytotoxicity. Furthermore, they showed that these NPs could overcome the BBB, probably thanks to their small size and the positive surface charge imparted by PEI. All the above allowed the author to propose C-dot/PEI as promising optical nanoprobes for live-cell imaging and as a good tool for drug delivery in brain diseases.

Li et al. [146] demonstrated that covalent conjugates of C-dots, Transferrin, and Doxorubicin (C-dot/Trans-Dox) exhibit a greater drug uptake than Doxorubicin (Dox) alone in several pediatric brain tumor cell lines, probably due to the high number of transferrin receptors on these tumor cells. Furthermore, they showed that C-dot/Trans-Dox were more cytotoxic than Dox alone, pointing to higher efficacy of the drug as conjugate.

Zheng et al. [147] synthesized a new type of C-dot (C-dot/Dex-Asp) targeting brain cancer gliomas via the direct pyrolysis of a mixture of Dextrose (Dex) and L-aspartic acid (L-Asp). The authors reported that C-dot/Dex-Asp exhibits excellent biocompatibility and tunable full-color emission, together with the significant capability of targeting C6 glioma cells without needing any extra targeting molecule. Moreover, after C-dot/Dex-Asp are injected through the mouse tail vein, the fluorescent signal detected at the glioma site is stronger than that recorded in the normal brain tissue. All the above confirms the ability of C-dot/Dex-Asp to penetrate the BBB freely and target the glioma tissue precisely, in contrast to the other C-dots synthesized from pure Dex, pure L-Asp, or a mixture of Dex and L-glutamic acid (L-Glu), which exhibited no or low selectivity for glioma.

Following these findings, Qiao et al. [148] optimized the ratio of Dex to L-Asp to improve the targeting properties of C-dot/Dex-Asp, their results indicating that C-dots prepared using a 7:3 molar ratio between Dex and L-Asp exhibit the greatest targeting ability toward C6 glioma cells.

Li et al. [135] developed C-dots that were covalently conjugated to Transferrin (Trans) or dye-labeled Trans (C-dot/Trans and C-dot/DyeTrans, respectively) to examine the potential of crossing the BBB via the Trans receptor-mediated delivery in the zebrafish model. In vivo results suggest that the Trans-conjugated C-dots can enter the CNS by overcoming the BBB, but C-dots alone cannot.

Hettiarachchi et al. [141] developed a triply-conjugated C-dots system (C-dot/Trans-Temo-Epi) for targeting glioblastoma, containing Trans and two anticancer drugs, i.e., Temozolomide (Temo) and Epirubicin (Epi). The authors compared its efficacy to the related doubly conjugated systems (C-dot/Trans-Epi, C-dot/Trans-Temo, C-dot/Temo-Epi) and the free-drug combinations against several glioblastoma cell lines. The obtained in vitro results indicated that the Trans-conjugated C-dots are able to reduce the cell viability considerably, compared to nanoparticles without Trans, and that C-dot/Trans-Temo-Epi possesses the greatest cytotoxicity.

Ruan et al. [149] employed a thermal treatment on Glycine (Gly) as the only starting material to produce C-dots (C-dot/Gly) and evaluated in vitro their cytotoxicity and cellular uptake and, in vivo, their tissue distribution and imaging properties. In vitro results obtained on C6 glioma cells demonstrated that C-dot/Gly possess low cytotoxicity and that their uptake is time- and concentration-dependent. In addition, the in vivo findings revealed a high accumulation of C-dots in the glioma tissue, which exhibited a stronger fluorescence intensity than normal brain one, indicating that these C-dots are indeed able to target glioma specifically.

Seven and co-workers [136] reported that C-dots (C-dot/Dex-Fluo) prepared from Dex and conjugated to Fluorescein (Fluo) are able to cross the BBB and carry a small molecular cargo to the CNS in two in vivo models, namely, zebrafish and rats, without the need for an additional targeting ligand.

Mintz et al. [150] developed two types of C-dots (C-dot/Try-urea and C-dot/Try-EDA) using Tryptophan (Try) and two different nitrogen dopants, e.g., urea and 1,2-ethylenediamine (EDA). The authors showed that these nanoparticles have low toxicity and can access the central nervous system of zebrafish, bypassing the blood-brain barrier via transporter-mediated endocytosis, exploiting the large neutral amino acid transporter 1 (LAT1). For the first time, this study demonstrates that tryptophan can be used as a precursor to yield self-targeting C-dots that do not need conjugation to other ligands.

In another study [151], Li et al. synthesized Large Amino Acid-Mimicking (LAAM) C-dots, bearing multiple paired α-carboxyl and amino groups, starting from 1,4,5,8-tetraminoanthraquinone and citric acid. The authors showed that LAAM C-dots are able to bind to the LAT1 and may be used for (i) near-infrared (NIR) fluorescence and photoacoustic imaging, (ii) targeted drug delivery to tumors with a high degree of specificity and efficiency, (iii) selectively imaging brain tumors, and (iv) the development of LAT1-utilizing prodrugs.

## 5. Conclusions

In the framework of brain tumors, with a particular focus on the open challenge on the delivery of drugs across to the blood-brain barrier, this review explores the recent advances in the use of drug delivery systems based on carbon dots. After a brief survey of the peculiarities of brain tumors and their current therapeutic management, the many exciting features of these recently discovered nanoparticles, leading to their applications as theranostic agents in cancer treatment, have been summarized. We have shown how their brightness and size-/composition-dependent photoluminescence, combined with their chemical stability and biocompatibility, have allowed their employment as fluorescent diagnostic tools. Various examples of the exploitation of the rich surface chemistry of C-dots have been illustrated, showing that their surface functional groups allow building on the anticancer and tumor-targeting features of these functional nanomaterials, also improving their ability to cross the BBB and reach the targeted neoplasms. The reported in vitro and in vivo studies strongly point out the bright potential of carbon dots as theranostic tools in future clinical strategies and could bring the development of more targeted therapies for brain tumor treatments.

## Figures and Tables

**Table 1:** Synthetic approaches to the production of C-dots.

Strategy	Synthetic Method	Refs
top-down	electrochemical synthesis	[68,69,70,71,72,73]
	chemical oxidation	[74,75,76,77,78,79]
	arc discharge	[61,80,81,82]
	laser ablation	[62,83,84,85,86]
bottom-up	ultrasound treatment	[87,88,89,114,115]
	hydrothermal treatment	[90,91,92,93,94,95]
	microwave-assisted synthesis	[96,97,98,99,100,101]
	pyrolysis	[102,103,104,105,106]

**Table 2:** Summary of C-dots-based drug delivery systems reported as being capable of crossing the BBB. Polyethyleneimine (PEI); Transferrin (Trans); Doxorubicin (Dox); Dextrose (Dex); L-aspartic acid (L-Asp); Temozolomide (Temo); Epirubicin (Epi); Glycine (Gly); Fluorescein (Fluo); Tryptophan (Try); 1,2-ethylenediamine (EDA); Large Amino Acid-Mimicking (LAAM).

C-Dots	Size (nm)	Drug Loaded	Ligand Attached	In Vitro/In Vivo	Administration Mode	Refs.
C-dot/PEI	2.6	None	None	Primary rat microvascular endothelial cells and astrocytes	Medium	[145]
C-dot/Trans-Dox	2–6	doxorubicin	transferrin	SJGBM2 and CHLA266 (pediatric brain tumor cells)	Medium	[146]
C-dot/Dex-Asp	2.3–2.5	None	None	C6 glioma cells/mouse	Medium/i.v. in tail vein	[147,148]
C-dot/Trans	5		transferrin	*Zebrafish*		[135]
C-dot/Trans-Temo-Epi	2.6–3.5	Temozolomide epirubicin	transferrin	SJGBM2, CHLA266, CHLA200 (pediatric brain tumor cells) and U87 (adult glioblastoma cells)	Medium	[141]
C-dot/Gly	<5	None	None	C6 glioma cells/mouse	Medium/Medium/i.v. in tail vein	[149]
C-dot/Dex-Fluo	2.4–2.5	None	None	Zebrafish and rat	i.v. into the heart and i.v. in tail vein	[136]
C-dot/Try-ureaC-dot/Try-EDA	9.0–10.8	None	None	Zebrafish	i.v. into the heart	[150]
LAAMC-dots	2.5	None	None	Mouse	i.v. in tail vein	[151]
